# Deep learning of quantitative ultrasound multi-parametric images at pre-treatment to predict breast cancer response to chemotherapy

**DOI:** 10.1038/s41598-022-06100-2

**Published:** 2022-02-10

**Authors:** Hamidreza Taleghamar, Seyed Ali Jalalifar, Gregory J. Czarnota, Ali Sadeghi-Naini

**Affiliations:** 1 Department of Electrical Engineering and Computer Science, Lassonde School of Engineering, York University, Toronto, ON Canada; 2 Department of Medical Biophysics, University of Toronto, Toronto, ON Canada; 3 Physical Sciences Platform, Sunnybrook Research Institute, Sunnybrook Health Sciences Centre, Toronto, ON Canada; 4 Department of Radiation Oncology, Odette Cancer Centre, Sunnybrook Health Sciences Centre, Toronto, ON Canada

**Keywords:** Biomedical engineering, Prognostic markers, Breast cancer, Cancer imaging, Cancer

## Abstract

In this study, a novel deep learning-based methodology was investigated to predict breast cancer response to neo-adjuvant chemotherapy (NAC) using the quantitative ultrasound (QUS) multi-parametric imaging at pre-treatment. QUS multi-parametric images of breast tumors were generated using the data acquired from 181 patients diagnosed with locally advanced breast cancer and planned for NAC followed by surgery. The ground truth response to NAC was identified for each patient after the surgery using the standard clinical and pathological criteria. Two deep convolutional neural network (DCNN) architectures including the residual network and residual attention network (RAN) were explored for extracting optimal feature maps from the parametric images, with a fully connected network for response prediction. In different experiments, the features maps were derived from the tumor core only, as well as the core and its margin. Evaluation results on an independent test set demonstrate that the developed model with the RAN architecture to extract feature maps from the expanded parametric images of the tumor core and margin had the best performance in response prediction with an accuracy of 88% and an area under the receiver operating characteristic curve of 0.86. Ten-year survival analyses indicate statistically significant differences between the survival of the responders and non-responders identified based on the model prediction at pre-treatment and the standard criteria at post-treatment. The results of this study demonstrate the promising capability of DCNNs with attention mechanisms in predicting breast cancer response to NAC prior to the start of treatment using QUS multi-parametric images.

## Introduction

Breast cancer is the most common cancer type and the foremost cause of cancer-related death among women^1,2^. In 2020, around 2.3 million new cases of breast cancer were diagnosed worldwide and it caused around 0.7 million deaths among females^1^. Locally advanced breast cancer (LABC) is an aggressive subtype of breast cancer that includes up to 20% of new cases each year^3^. LABC is often identified with tumors greater than 5 cm in size and possibly with skin and/or chest wall involvement. Moreover, LABC includes patients diagnosed with inflammatory breast cancer or multiple positive axillary lymph nodes^3,4^.

Patients diagnosed with LABC suffer from high risk of relapse and metastasis with a local recurrence rate of about 48% in 5 years^5^. With availability of different systemic and targeted regimens, neoadjuvant chemotherapy (NAC) followed by surgery is currently considered as the standard treatment for LABC patients^6–9^. In some cases, the surgery is followed by adjuvant radiation and/or hormonal therapies to reduce the risk of cancer recurrence^4,6^. Although response to NAC has demonstrated a high correlation to the patient survival, complete pathological response is limited to less than 30% of the patients, with about 30% of the patient do not even partially respond to NAC^3,4,6,10–14^. To determine the tumor pathological response to NAC, post-surgical histopathology is considered as the standard approach^6–9^. However, post-surgical evaluations cannot be used for adjusting the NAC or switching to salvage treatment.

Currently, monitoring tumor response to NAC mostly relies on physical examination or standard anatomical imaging to assess the changes in tumor size. The main limitation of these methods is that detectable changes in tumor dimensions usually become apparent after several months of therapy, and in some cases a measurable change may not become evident on imaging despite a pathological response to NAC^15^. Early prediction of tumor response to NAC can permit therapy adjustments by modifying the regimen, dose, and/or sequence of treatment options, switching to more effective treatments or even salvage therapies before it is potentially too late for individual patients^16,17^. A personalized strategy for LABC treatment is anticipated to improve tumor response to neoadjuvant therapies, spare patients from unnecessary side effects of ineffective treatment, and improve their overall survival and quality of life.

Ultrasound is a portable, rapid and cost-efficient imaging modality that can be applied to characterize tissue physical properties without injection of any exogenous contrast agents. In particular, quantitative ultrasound (QUS) techniques have been introduced to derive quantitative measures of tissue biophysical properties that are independent of instrument settings, with a lower level of dependence to the operator^18^. Quantitative ultrasound spectral analysis techniques examine the frequency dependence of the ultrasound radiofrequency (RF) signal backscattered from the underlying tissue which can be used for tissue micro-structure characterization^18^. The QUS parameters derived from the analysis of normalized power spectrum of RF signal including the mid-band fit (MBF), spectral slope (SS), spectral 0-MHz intercept (SI), effective scatterer diameter (ESD) and effective acoustic concentration (EAC) have shown promises in detecting and characterizing malignancies, examination of liver tissues and detecting cardiovascular disease^19–25^.

It has been shown that QUS spectral parameters can detect tumor cell death induced by various anti-cancer-therapies^26–29^. Also, several studies have demonstrated that the hand-crafted features derived from the QUS parametric maps can be used to predict and monitor breast cancer response to neoadjuvant chemotherapy before or within weeks after the start of treatment, with a high correlations to clinical and pathological response identified at the end of treatment^30–33^. For example, it has been demonstrated that textural features of QUS spectral parametric maps have higher correlations to histological tumor cell death in response to chemotherapy in comparison with QUS mean-value parameters^34^. Further, A few studies have revealed the potential of the textural features of QUS parametric images in predicting LABC tumor response to NAC as early as 1 week after starting the treatment^35–37^. In a recent study, Tadayyon et al. have demonstrated that using the QUS hand-crafted features derived from both the tumor core and its margin could improve the performance of tumor response prediction before starting the treatment^38^.

The deep learning approaches have recently been investigated in different applications of medical image analysis^39,40^. Such methodologies can potentially remove the process of extracting carefully designed hand-crafted features from images required for conventional machine learning techniques. Instead, the deep learning frameworks optimize their data-driven feature maps during the iterative training procedure^41^. In this context, a few studies have been conducted on adapting deep convolutional neural networks (DCNN) for NAC response prediction in breast cancer patients using magnetic resonance imaging (MRI)^42–44^. Moreover, few studies have explored the potential of DCNNs in analyzing ultrasound images of breast tumors for cancer classification. For example, Byra et al. have demonstrated the potential of convolutional neural networks for breast lesion classification using Nakagami parametric images^45^. To our knowledge, no previous study has explored the efficacy of deep learning techniques with QUS multi-parametric images for therapy response prediction.

In this study, the effectiveness of DCNN methodologies on QUS spectral multi-parametric images has been investigated to predict LABC response to NAC before the start of treatment. The QUS spectral parametric images were generated using the ultrasound data acquired from 181 LABC patients at pre-treatment. The patient responses to NAC were identified after their surgery using the standard clinical and pathological criteria, and used as the ground truth to evaluate the performance of the prediction models. The dataset was randomly partitioned into a training set and an independent test set. Different DCNN architectures including RAN^46^, and ResNet^47^ were investigated for feature extraction in the developed framework. In a set of experiments, the feature maps were extracted from the tumor core and the core and its margin. After averaging the features on different tumor cross-sections, a fully connected network was utilized for response prediction. The results demonstrate that the developed model with the RAN architecture for extracting feature maps from the expanded parametric images of the tumor core with margin had superior performance with an AUC of 0.86 on the independent test set. The Kaplan–Meier survival analyses show that the patients predicted as the responders using this model demonstrated a significantly better survival rate in comparison with those predicted as non-responders.

## Materials and methods

### Study protocol

This study was conducted following the guidelines and regulations in accordance with institutional research ethics board approval from Sunnybrook Health Sciences Centre (SHSC), Toronto Canada. The study was open to all women aged 18–85, diagnosed with LABC and planned for NAC followed by surgery. After obtaining written informed consent, 181 eligible patients were recruited for the study. A core needle biopsy was done for each patient to confirm the cancer diagnosis and grade the tumor. The initial tumor size was determined for each patient using the magnetic resonance (MR) images of the affected breast. Ultrasound B-mode images and radiofrequency (RF) data were acquired from the patients (in supine position with arms above the head) before the start of NAC, following a standardized protocol. Three experienced sonographers were responsible for ultrasound data acquisition. For NAC, 62.9% of the patients received doxorubicin, cyclophosphamide followed by paclitaxel/docetaxel (AC-T/D), 32.6% were treated with 5-fluorouracil, epirubicin, cyclophosphamide followed by docetaxel (FEC-D), and 4.5% with paclitaxel and cyclophosphamide (TC). The patients with HER2+ tumors also received tratuzumab. Patients were followed up to 10 years after their treatment and their clinical data were recorded for survival analysis. Out of the 181 patients, about 30% (n = 50) were randomly selected through a stratified random sampling and kept unseen as an independent test set, and the remaining patients (n = 131) were considered as the training set and used to develop and optimize the predictive models.

### Clinical and pathological response evaluation

In keeping with the institutional guidelines, all patients had breast surgery after completing their neoadjuvant chemotherapy. Before surgery, the residual tumor size was determined using MRI. Standard histopathology was performed on the surgical specimens to assess the pathological response of tumor to NAC. The specimens were stained with hematoxylin and eosin (H&E) and prepared when possible on whole-mount 5″ × 7″ pathology slides. The mounted slides were digitized using a confocal scanner (TISSUEscope, Huron Technologies, Waterloo, ON). A board-certified pathologist who remained blinded to the study results examined all pathology samples. A modified response (MR) grading system based on response evaluation criteria in solid tumors (RECIST)^48^ and histopathological criteria^38,49^ was used to categorize the patients into two groups of responders and non-responders, as before^50^. In the MR grading system, the MR score is defined as follows: MR 1: no reduction in tumor size; MR 2: less than 30% reduction in tumor size; MR 3: between 30 and 90% reduction in tumor size or a very low residual tumor cellularity determined histopathologically; MR 4: more than 90% reduction in tumor; MR 5: no evident tumor and no malignant cells identifiable in sections from the site of the tumor (ductal carcinoma in situ may be present). In this study, patients with a MR score of 1–2 (less than 30% reduction in tumor size) were considered as non-responders, and patients with a MR score 3–5 (more than 30% reduction in tumor or very low residual tumor cellularity) were determined as responders. In keeping with this, 138 and 43 patients were identified as responders and non-responders, respectively.

### Ultrasound data acquisition

Ultrasound RF data were acquired utilizing an RF-enabled Sonix RP system (Ultrasonix, Vancouver, Canada) and an L14-5/60 transducer. The transducer operated at the center frequency of ~ 6 MHz with a − 6 dB bandwidth of 3–8 MHz. The RF data were acquired with a sampling frequency of 40 MHz and digitized with a 16-bit resolution. For each tumor, the ultrasound data were acquired at four to seven image planes across the breast with approximately 1 cm intervals. The focal depth was set at the center of the tumor depending on the individual patient circumstances. The breast region for ultrasound scanning was specified by an oncologist who determined the acquisition scan planes via a physical examination of the patient. The image size along the lateral and axial directions was 6 cm and 4–6 cm, respectively.

### QUS parametric map generation

For generating the parametric images, QUS spectral analyses were performed in conjunction with a sliding window analysis (described below) to derive MBF, SI, ESD, and EAC parameters^22,23^. The mean power spectrum was obtained by averaging over the Fourier transform of the Hanning-gated RF data calculated for every scan line of the analyzed window. The average power spectrum was normalized using a reference phantom method to remove the effects of the system transfer function and transducer beam-forming^51,52^. The reference phantom was composed of 5–30 μm diameter glass beads embedded in a homogeneous background of microscopic oil droplets in gelatin (Medical Physics Department, University of Wisconsin, USA). The reference phantom had an attenuation coefficient of 0.576 dB/MHz.cm and a speed of sound parameter of 1488 m/s. The MBF and SI parameters were estimated using a linear regression analysis within the − 6 dB bandwidth of the transducer^22,53,54^. The ESD and EAC parameters were derived by fitting a spherical Gaussian form factor model to the estimated backscatter coefficient^55,56^.

To generate the QUS parametric maps for each tumor, the tumor core was manually outlined by trained staff under the supervision of expert oncologists on each scan plane using the associated B-mode image. The tumor margin contour was automatically generated with a thickness of 5 mm around the core, based on the observations of a previous study^38^. The parametric maps were generated for all imaging planes of the tumor using a sliding window analysis throughout the entire region of interest (tumor core and margin) with windows of size 2 mm × 2 mm and 95% overlap in both lateral and axial direction, where the calculated parameter for each window was assigned to its center. The sliding window size was selected such that it covers sufficient ultrasound wavelengths in the axial direction for spectral analysis while preserving texture in generated parametric maps, with an overlap size to obtain isotropic pixels^57,58^.

### Deep learning model

**Figure 1:**
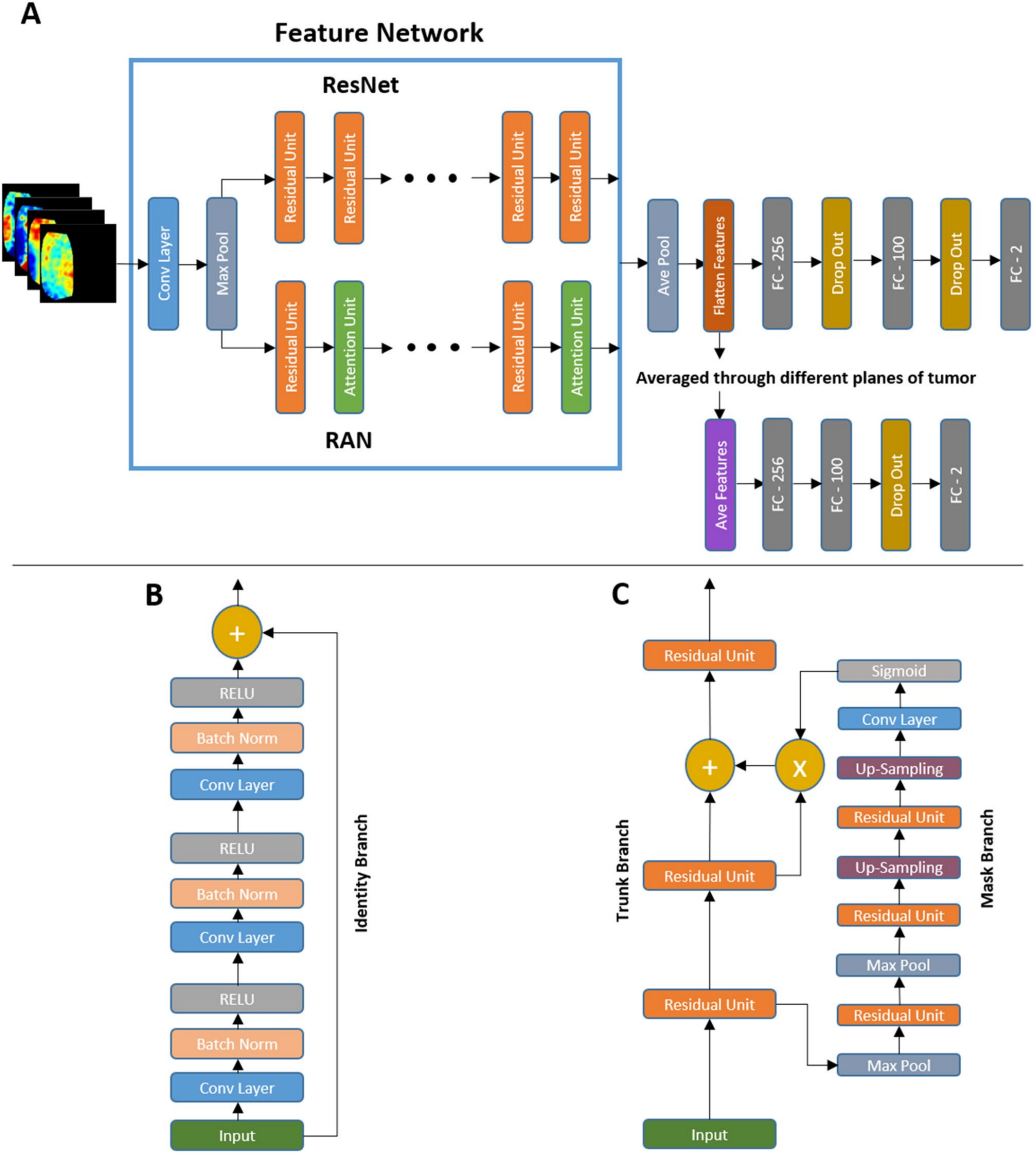
Scheme of the developed deep learning framework for response prediction, demonstrating the feature and predictive networks (**A**), the residual module (**B**), and the attention module (**C**).

The scheme of the deep learning framework developed in this study for response prediction is shown in Fig. 1. The framework consists of two cascaded networks. The first network is a DCNN, with several convolutional layers as its backbone, adapted to extract the optimal feature maps from the QUS parametric images and is called the feature network in this paper. Two main architectures including a modified residual network version 101 (ResNet)^47^ and a modified residual attention network version 56 (RAN)^46^, were investigated in this study as the backbone of the feature network. Figure 1 demonstrates the fully connected layers after the convolutional layers (backbone) in the feature network that are applied in training this network on single parametric images to extract the optimal feature maps for response prediction. The figure also depicts the adapted ResNet and RAN architectures with their residual and attention modules. In the residual module applied in the ResNet architecture, the convolutional layers could be skipped through the identity branch. This strategy permits a more efficient training of very deep networks such as ResNet to achieve an improved performance on unseen samples. In the attention module applied in the RAN architecture, the trunk branch determines the information that can be passed through the network, whereas the mask branch determines the amount of information from the trunk branch that should be passed. Therefore, the module is able to pass the important information with higher weights and reduce the effect of less-important information in the network’s output.

As shown in the Fig. 1, the optimized feature maps obtained from the feature network are averaged over all parametric images associated with each tumor, and subsequently used in a second fully connected network (predictive network) adapted for response prediction at the patient level (described further below). The predictive network consists of two fully-connected layers with an input layer with the same size as the flatten feature vector (256), a middle layer with 100 neurons, and a softmax layer at the end with an output size of two to predict the probability of the response categories (responder vs. non-responder) for each patient. Drop-out layers have been added after the first and second layer of this network to avoid overfitting and enhance its generalization performance.

### Preprocessing and model training

Before training the models, the parametric images were preprocessed and adjusted for the convolutional model. About 25% (31 patients) of the training set was randomly selected as a validation set to optimize the network hyperparameters. The parametric images were resampled to the size of 512 × 512 pixel. Then the pixel values in the parametric images of the training set were normalized to (0 1) to facilitate the training convergence. The training set normalization parameters were used for normalizing the validation and test sets. In order to improve the network training and alleviate the problem of having a relatively small training dataset, data augmentation was applied on the training set. For augmenting the training data, flipping horizontally and shifting both horizontally and vertically (maximum shift: 30% of image size) were stochastically applied.

In the first step of model training, the feature network was trained to generate the optimal feature maps for single QUS parametric images. For training the feature network, parametric images of the training set were fed into the network while different imaging planes of each tumor were considered as independent inputs with the tumor response as their output. The optimal feature maps for each imaging plane were acquired by feeding its corresponding parametric images into the trained feature network. For each patient, the optimal feature maps were calculated for all 2D imaging planes of the tumor, flatten to a 1D vector, and subsequently averaged over the entire tumor volume to obtain an averaged feature vector with size of 256 × 1 that was used in the predictive network. This strategy was applied to standardize the input size of the networks for different tumors with various sizes and, consequently, different number of QUS parametric images. The predictive network was trained using the averaged feature vectors associated with the patients in the training set, and evaluated over the independent test set for response prediction. For training the networks, the cross entropy was used as the loss function, with a cost weight ratio of C:1 (C ≥ 1; optimized as described below) for non-responders to responders to account for the unbalance in the dataset. The network hyperparameters including the dropout rate (range 0.3–0.7), width of the hidden fully connected layers (range 50–300), learning rate (range 0.1–0.00001), cost weight (range 1 ≤ C ≤ 10), and batch size (range 4–16) were optimized using the validation set. Preliminary experiments were conducted using the validation set to select the network training optimizer among the Adam and stochastic gradient descent (SGD) methods, where the Adam optimizer was selected and applied^59^. The optimal hyperparameters for training the models were as follows: dropout rate = 0.5, learning rate = 0.0001, cost weight = 5, batch size = 8. Early stopping was used to avoid overfitting by monitoring the network’s performance on the validation set during the training process.

### Response prediction and risk assessment

In different experiments, the QUS multi-parametric images (MBF, SI, ESD, and EAC) of the tumor core, as well as the core and its margin were investigated, as the inputs to the DCNN framework and their performance were compared in response prediction. The deep learning models with different feature networks were trained and optimized using the training set. The performance of the optimized models was evaluated on the independent test set using the accuracy, sensitivity, specificity, and the ROC analysis. In this study, sensitivity refers to the ratio of the non-responses that were predicted as non-responder, and specificity refers to the ratio of the responding patients correctly predicted as responder by the model. A prediction difference analysis (PDA) was performed to visualize the importance of different regions of the input QUS parametric images to the network’s decision^60^. In each iteration of the modified PDA procedure applied in this study, a small patch (8 × 8 pixel with 50% overlap between adjacent patches) of one of the input parametric images was occluded (pixel values were set to zero). The absolute change in the model’s prediction (output probability) was then calculated compared to the case of inputting the original parametric images, and considered as the impact of the occluded patch on the network’s decision. The PDA maps were generated for each input parametric image by sliding the occluding patch over the image and assigning the estimated impact to its center.

The efficacy of the developed predictive models in differentiating the LABC patients with different recurrence-free survival was assessed through Kaplan–Meier survival analysis. The survival curves were generated for the responders and non-responders identified based on each model’s prediction at pre-treatment, and at post-treatment based on the clinical and histopathological criteria. A long-rank test was applied to assess for statistically significant differences between the survival curves of the two response cohorts obtained in each experiment.

## Results

Table 1 presents the clinical and histopathological characteristics of the participating patients. The patients had an average initial tumor size of 5.2 cm, and an average residual tumor size of 2.5 cm at the end of treatment. Using the MR grading system, 76.2% and 23.8% of the patients were identified as responders and non-responder, respectively, at the end of treatment.

**Table 1:** Patients’ characteristics.

Data set	All	Training	Test
Characteristic	Mean ± SD/Percentage		
Age	50.6 ± 11.5 years	51.2 ± 11.5 years	49.2 ± 11.4 years
Initial tumor size	5.2 ± 2.7 cm	5.3 ± 2.7 cm	5.1 ± 2.7 cm
Residual tumor size	2.5 ± 3.4 cm	2.8 ± 3.7 cm	1.9 ± 2.3 cm
**Histology**			
Invasive ductal carcinoma	90.3%	89.8%	91.7%
Invasive lobular carcinoma	3.4%	4.6%	0.0%
Invasive metaplastic carcinoma	6.3%	5.6%	8.3%
**Tumor grade**			
Grade I	10.6%	12.1%	10%
Grade II	38.8%	36.4%	45%
Grade III	50.6%	51.5%	45%
**Molecular features**			
ER +	63.4%	62.5%	64.4%
PR +	54.7%	55.5%	51.1%
HER2 +	34.3%	30.5%	46.7%
Triple negative	24.4%	26.6%	17.8%
ER + /PR + /HER2 +	18.6%	18.0%	20.0%
ER + /PR + /HER2-	33.7%	35.9%	26.7%
ER-/PR-/HER2 +	10.5%	9.4%	15.5%
**NAC**			
AC-T/D	62.9%	63.2%	62%
FEC-D	32.6%	31.2%	36%
TC	4.5%	5.6%	2%
**Therapy response**			
Responder	76.2%	74.8%	80%
non-responder	23.8%	25.2%	20%

**Figure 2:**
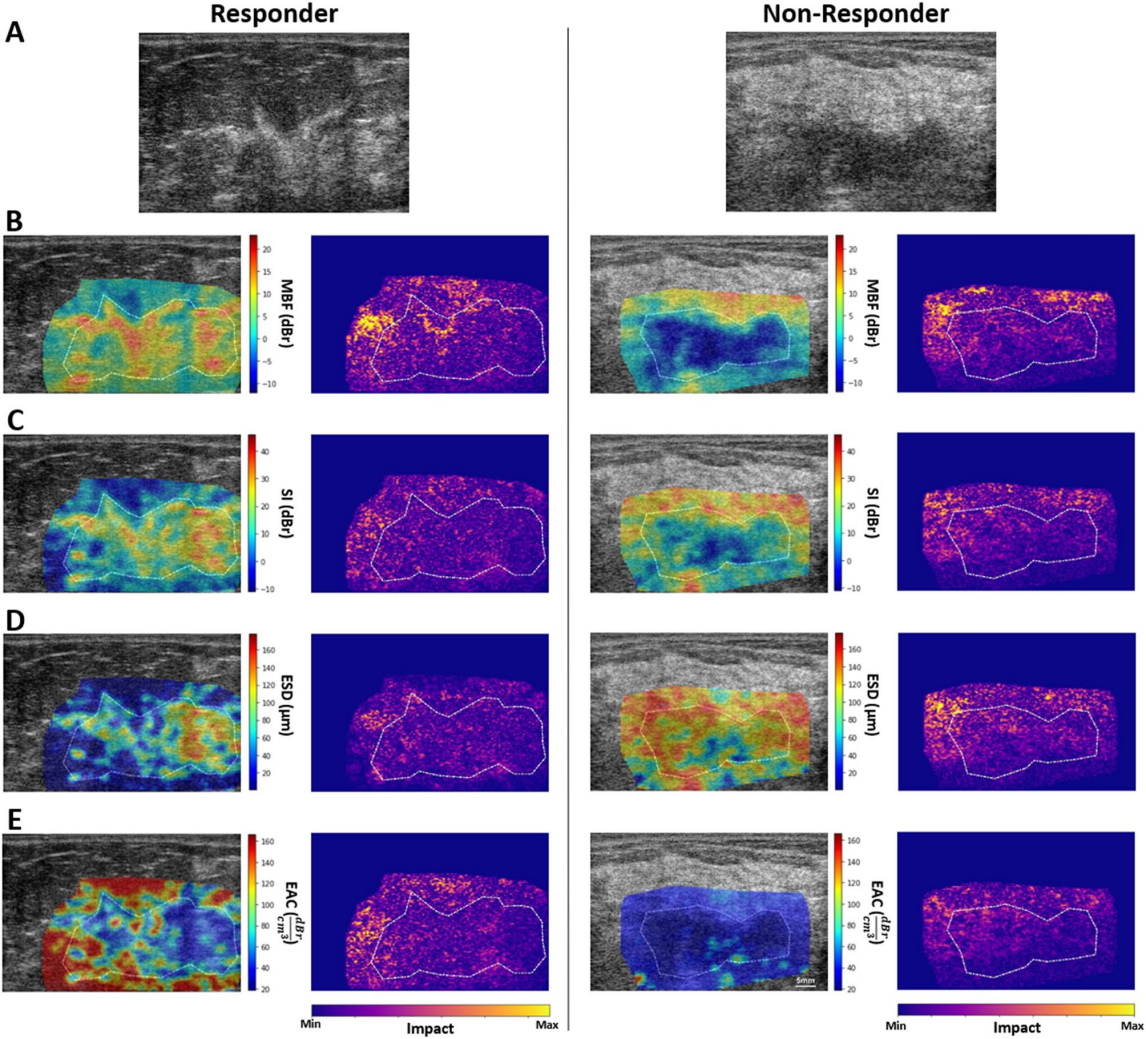
Ultrasound B-mode images (**A**), and parametric overlays of MBF (**B**), SI (**C**), ESD (**D**), and EAC (**E**) on B-mode images acquired at pre-treatment from a representative responder and non-responder to NAC, and the associated PDA maps visualizing the level of impact of different regions in each parametric image on the network’s decision (model 4 in Table 2). The tumor core has been outlined with white dashed line.

**Figure 3:**
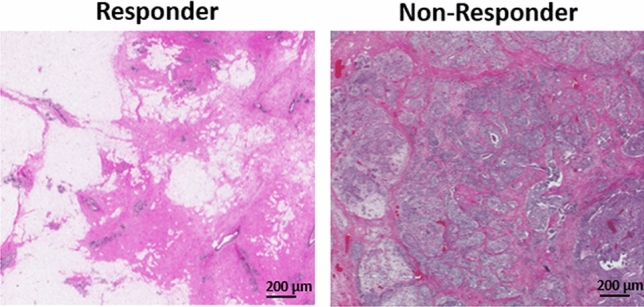
Histopathology images of surgical specimens obtained from representative patients.

Figure 2 demonstrates QUS parametric maps of MBF, SI, ESD, and EAC overlaid on the ultrasound B-mode images obtained from representative responding and non-responding patients, respectively. As observed in these representative images, the QUS parametric maps associated with the responding and non-responding patients demonstrated different mean and spatial pattern of pixel values within the tumor core and margin. The figure also shows the PDA maps associated with these parametric images, visualizing the relative impact of different regions in each image to the network’s decision for response prediction. Figure 3 demonstrates H&E stained histopathology images of the surgical specimens acquired from representative responding and non-responding patients. In responding patients, minimal tumor cellularity usually remained within the tumor bed after chemotherapy, as evident in the histopathology slides. In contrast, histopathology images of the non-responding patients typically indicated large areas of residual disease with minimal chemotherapy effects.

**Figure 4:**
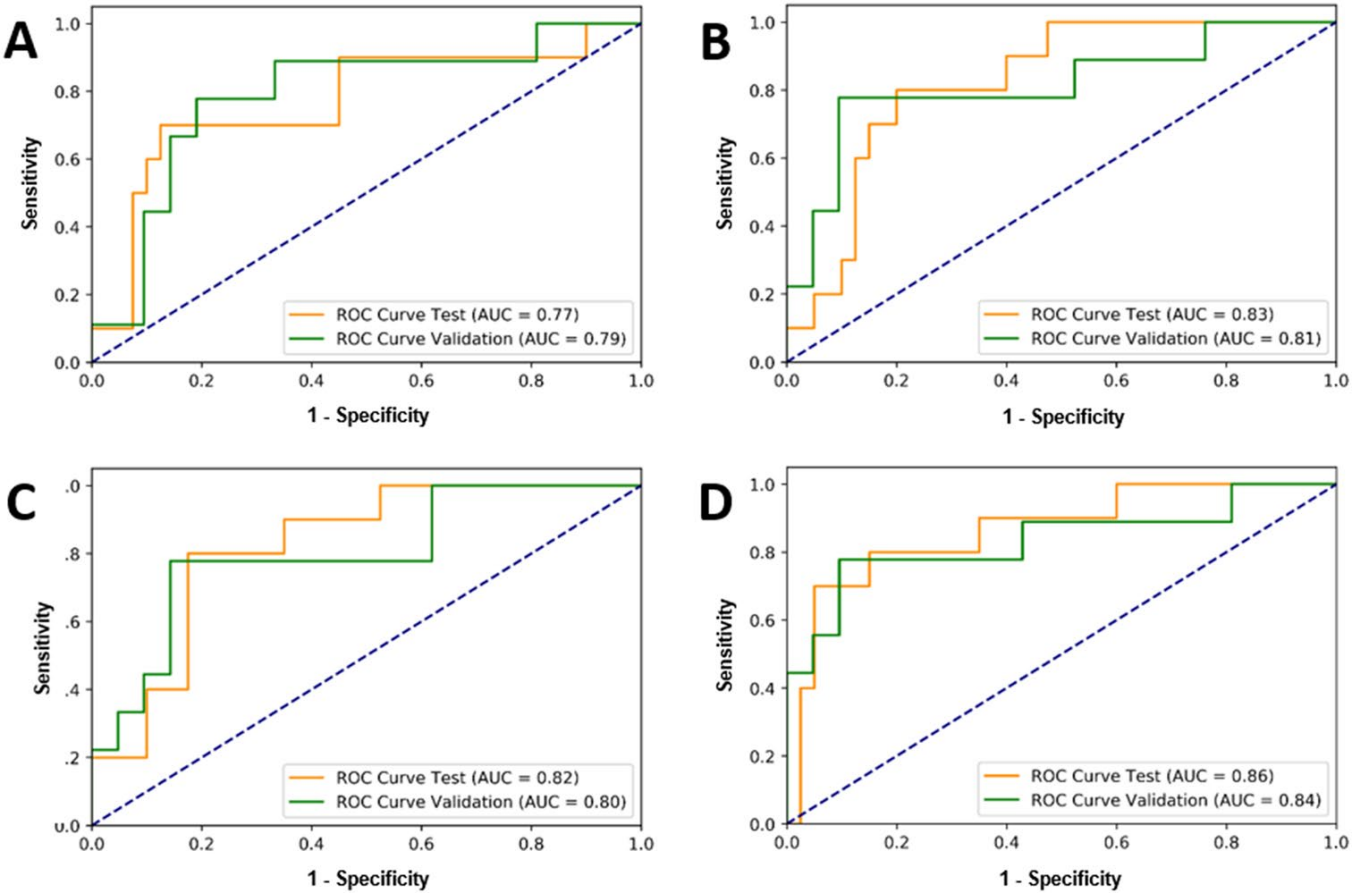
ROC curves generated for responding and non-responding patients in the validation set and independent test set identified at pre-treatment using the predictive models 1–4 in Table 2 (**A**–**D**).

Table 2 presents the results of response prediction in different experiments on the validation and independent test sets. The ROC curves associated with the validation and test sets for different predictive models are demonstrated in Fig. 4. Using the ResNet architecture as the model’s backbone to extract feature maps from the parametric images of the tumor core resulted in an AUC of 0.77 on the independent test set. Extending the input parametric images to include both the tumor core and its margin improved the AUC of this model to 0. 83.

**Table 2:** Results of response prediction on the validation and independent test sets with different models.

Model	Feature network	Input parametric maps	Validation set				Test set			
			Acc (%)	Spec (%)	Sen (%)	Loss	Acc (%)	Spec (%)	Sen (%)	AUC
1	ResNet	Core	77 ± 15	76	78	0.27	80 ± 11	82.5	70	0.77 ± 0.12
2	ResNet	Core + margin	86 ± 12	90	78	0.17	82 ± 11	85	70	0.83 ± 0.10
3	RAN	Core	83 ± 13	86	78	0.22	80 ± 11	80	80	0.82 ± 0.11
4	RAN	Core + margin	86 ± 12	90	78	0.16	88 ± 9	92.5	70	0.86 ± 0.10

*Acc* Accuracy ± 95% confidence interval, *Spec* specificity, *Sen* sensitivity, *AUC* area under the ROC curve ± 95% confidence interval.

Applying the extracted features from the parametric images of the tumor core using the RAN architecture resulted in an accuracy of 80%, and an AUC of 0.82 on the independent test set. Similar to the model with the ResNet architecture as the feature extractor, the overall performance of this model improved by extending the input parametric images to include the tumor margin. In particular, this model resulted in the best prediction performance with an accuracy and AUC of 88%, and 0.86, respectively, on the independent test set. All models demonstrated a relatively similar performance on the validation and test sets, implying a good generalizability of the trained models on never seen samples.

**Figure 5:**
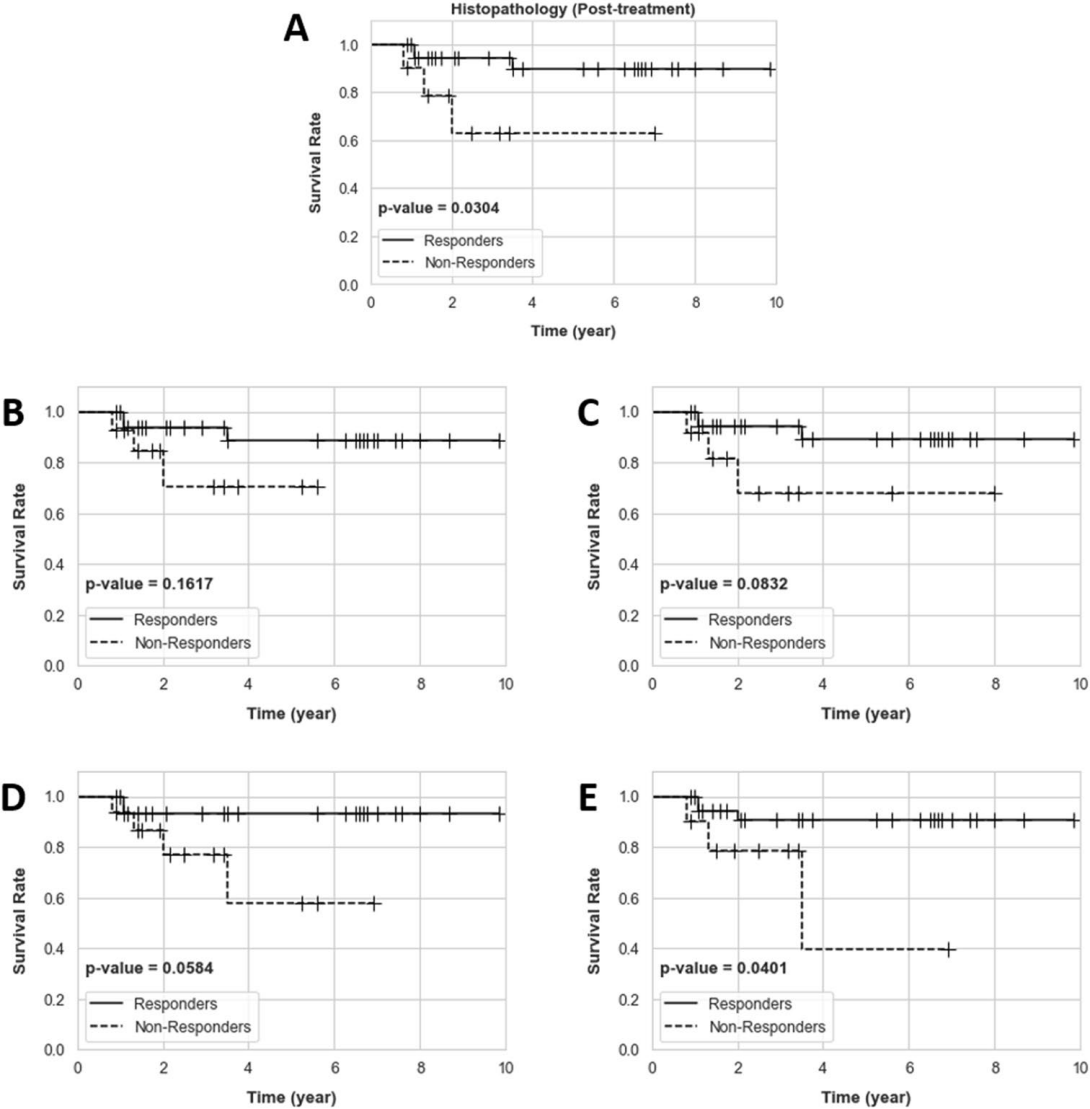
Recurrence-free survival curves for the two patient cohorts in the independent test set. The responders and non-responders were identified at post treatment based on the clinical and histopathological criteria (**A**), and at pre-treatment using the predictive models 1–4 in Table 2 (**B**–**E**).

Figure 5 presents the 10-year recurrence-free survival curves for the responders and non-responders identified based on the clinical and histological criteria at post treatment, and at pre-treatment using the four predictive models presented in Table 2. The survival analysis demonstrated a statistically significant difference (*p* value = 0.030) between the survival curves of the responders and non-responders identified at post-treatment. Among the response cohorts predicted at pretreatment, the ones identified using the predictive models with the RAN as their feature network demonstrated a statistically significant difference or approaching significance. Specifically, whereas the model that input the parametric images of the tumor core approached a significant difference (*p* value = 0.058), the one with the input parametric images extended to the tumor margin demonstrated a statistically significant difference between the survival of the two predicted cohorts (*p* value = 0.040). The response cohorts identified by the other two models at pre-treatment did not show a significant difference in survival.

## Discussion and conclusion

In this study, a deep learning methodology was proposed and investigated for predicting breast tumor response to NAC using the QUS parametric images acquired at pre-treatment. In the proposed framework, a DCNN architecture is utilized to extract optimal feature maps from QUS multi-parametric images. The flatten feature vectors associated with different cross-sections of a tumor are averaged over the entire tumor volume. A fully-connected network is subsequently applied for response prediction, using the average feature vectors. The performance of the framework was investigated in a set of experiments using two different DCNN architectures including the ResNet and RAN as the backbone feature extractor, and the QUS parametric images of the tumor core, and the tumor core with margin as the input. The best performance in response prediction was achieved with the RAN architecture and the extended parametric images of the tumor core and margin, with an AUC of 0.86 on the unseen test set. Long-term survival analyses demonstrated statistically significant differences between the survival of the responders and non-responders identified at pre-treatment based on this model’s prediction, and at post-treatment based on the standard clinical and pathological criteria.

A number of previous studies have investigated the potential of QUS multi-parametric imaging in predicting and monitoring response of breast cancer tumors to NAC^38,61^. In those studies, handcrafted features such as the statistical and textural features of the QUS parametric maps were analyzed and applied for response prediction using traditional machine learning methods. With predefined hand-crafted features, the feature extraction process is easier to understand and analyze. But these features only carry limited information based on their definition. Further, carefully determining and defining optimal discriminatory features for each specific application is crucial in this approach but is not always feasible. In this study, we investigated the potential of DCNN architectures for extracting optimal features from QUS multi-parametric images automatically through multiple convolutional layers. The advantage of using deep learning model for feature extraction is that the model learns during the training process to extract the important information while considering the whole image. As the PDA maps generated for the QUS parametric images implied, different regions of these parametric maps contribute substantially to the model’s prediction, albeit with varying levels.

It has been shown that intratumor heterogeneity is an important factor in responsiveness of tumors to cancer therapy, with several studies demonstrated the value of imaging-based quantitative features of tumor heterogeneity for response prediction^23,62–65^. The results obtained in the study here using the DCNN feature extractors are in agreement with the observations of those studies. Specifically, the features maps extracted from the QUS multi-parametric images are computed using multiple layers of convolutional filters throughout a DCNN architecture and can quantify the spatial heterogeneity within multi-channels of the QUS images simultaneously. The results of different experiments performed in this study demonstrated that extending the input QUS parametric images to include the tumor margin in addition to the core enhances the performance of the model in response prediction. Further, whereas the PDA maps demonstrated considerable contribution of various regions of the QUS parametric images to the network’s decision, the impact of the margin areas on the model’s prediction was highlighted particularly in the MBF and EAC parametric images. These results are in agreement with observations of the previous studies in which the imaging-based characteristics of tumor margin were shown important in different diagnostic and prognostic applications^38,65–67^.

The RAN architecture demonstrated a better performance compared to the ResNet in deriving optimal feature maps from QUS parametric images for response prediction. Both architectures use residual modules to overcome the overfitting throughout their very deep layers. The RAN architecture also utilizes attention modules that potentially facilitate feature optimization. Specifically, the attention module enables the network to focus on significant and influential regions of images while extracting the feature maps. The observations of this study imply that the attention modules in the RAN architecture have been successful in identifying the important regions of the QUS parametric maps for response prediction.

A potential limitation associated with this study is the relatively small dataset that was available to train and optimize the models. Training large DCNN models on small datasets may result in overfitting and lack of generalizability. Effective approaches including network regularization through drop-out layers and early training stopping was applied in this study to reduce the chance of model overfitting and improve its generalizability. An independent unseen test set was applied to evaluate the model’s performance, where the results demonstrated a good generalizability on never seen samples. Availability of larger datasets in future would permit more comprehensive training of the models and more rigorous evaluation of their prediction performance for new patients. In terms of NAC, the majority of the patients in this study (95.5%) were treated with either AC-T/D or FEC-D. The performance of the response prediction models in this study was relatively similar for the patients treated with different NAC regimens with the number of incorrect predictions proportional to the number of patients receiving each regimen. Nevertheless, response to different NAC regimens may be different for a patient. Future stratified analysis on larger datasets with separate models for different NAC regimens and possibly various molecular subtypes may result in more robust predictive models. Such models can also facilitate future randomize clinical trials to investigate the adjustment of NAC regimen for patients with low likelihood of response to the standard treatment. In conclusion, this study demonstrated that DCNN models can be adapted in the context of quantitative imaging for therapy response prediction. The results indicated a better performance of the attention-guided convolutional networks in deriving optimal quantitative features form QUS multi-parametric images. The deep learning models developed in this study could predict the survival-linked response of LABC patients to NAC before starting the treatment with a high accuracy. Predicting the outcome of NAC at pre-treatment would help the clinicians to adjust ineffective treatment regimens for individual patients. The results obtained in this study are promising and encourage further investigations using other DCNN architectures with the data acquired from larger (multi-institutional) cohorts of patients to evaluate the robustness of the methodologies in clinic.

## Supplementary Information

Supplementary Information.
