# Supplementary material for: Deep learning of quantitative ultrasound multi-parametric images at pre-treatment to predict breast cancer response to chemotherapy
Source: Sci Rep. 2022 Feb 10;12:2244. doi: 10.1038/s41598-022-06100-2 (PMC8831592; doi:10.1038/s41598-022-06100-2)
Supplement: Supplementary file 1 — Supplementary Information. [file 41598_2022_6100_MOESM1_ESM.pdf]

Supplementary Materials

Deep Learning of Quantitative Ultrasound Multi-Parametric Images at Pre-treatment to Predict Breast Cancer Response to Chemotherapy

Hamidreza Taleghamar[(1)], Seyed Ali Jalalifar[(1)], Gregory J. Czarnota[(2,3,4)], and Ali Sadeghi-Naini[(1,2,3,4)]

(1) Department of Electrical Engineering and Computer Science, Lassonde School of Engineering, York University, Toronto, ON, Canada

(2) Department of Medical Biophysics, University of Toronto, Toronto, ON, Canada

(3) Physical Sciences Platform, Sunnybrook Research Institute, Sunnybrook Health Sciences Centre, Toronto, ON, Canada

(4) Department of Radiation Oncology, Odette Cancer Centre, Sunnybrook Health Sciences Centre, Toronto, ON, Canada

Network Architectures

1. Residual Network version 101 (ResNet)

The deep residual network was introduced by He *et al.* [1] in 2015. In this architecture, shortcut connections are utilized to perform identity mapping, and as a result, outputs of previous layers are added to the outputs of the stacked layers. For implementing the shortcut connections, a residual module (Figure 1.B of the paper) is applied. In this architecture, the identity shortcut connections add neither extra parameters nor computational complexity.

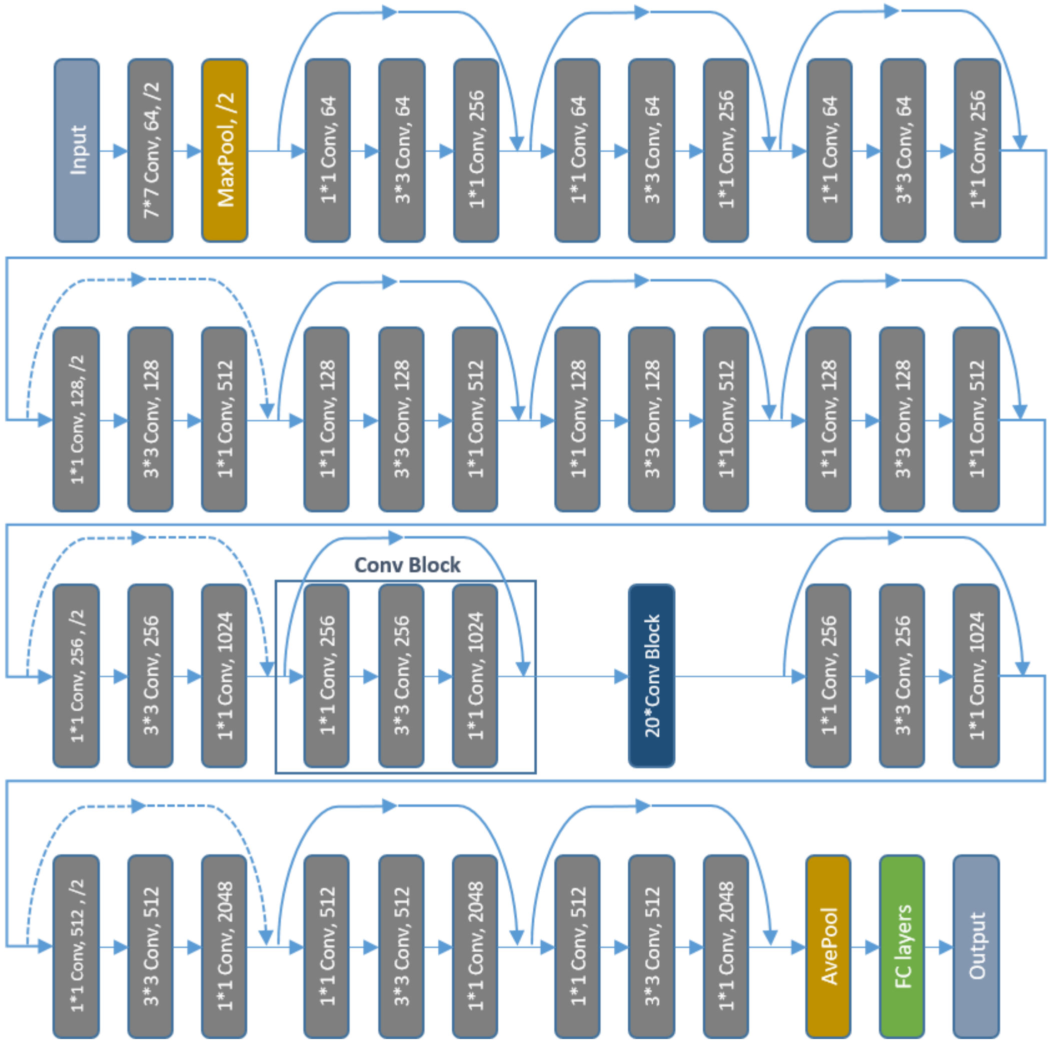

Figure S1. ResNet101 architecture.

The ResNet101 architecture is demonstrated in Figure S1. The identity shortcuts can be directly used when the dimensions of the input and output are the same (solid line shortcuts in Figure S1). When the spatial size decrease (dotted line shortcuts in Figure S1) the projection shortcut is used to match the output dimensions by applying 1×1 convolutions with a stride of 2.

The ResNet framework was introduced to address the degradation problem in very deep neural networks: as network depth increases in very deep NNs, accuracy becomes saturated and then rapidly decreases. A main problem with a very deep network is that the gradient could not reach the early layers and is lost due to backpropagation through several layers. Residual blocks allow forward propagation to skip layers, and the gradient be back-propagated to initial layers without vanishing. Also, because the shortcut connections add no extra parameters or complexity to the computation, they do not increase the computational cost for training.

2. Residual Attention Network version 56 (RAN)

The residual attention network [2] is a convolutional network that uses an attention mechanism in a "very deep" architecture, inspired by the attention mechanism in deep neural networks. The RAN includes several stacked attention modules, each of which generates attention-aware features.

Figure 1.C of the paper depicts the attention module utilized in the RAN. In this attention module, instead of simply outputting dot production of features with mask, a scheme for attention residual learning has been proposed. As a result, the output of attention module could be presented as follows:

$H(x) = (1 + M(x)) * F(x)$ (Eq. 1)

where M(x) and F(x) are outputs of mask branch and trunk branch, respectively. In this architecture, the trunk branch is in charge of feature processing. The output of the mask branch is utilized to regulate the neurons of the trunk branch. In the mask branch, max pooling is performed numerous times from the input to enlarge rapidly the receptive field after a number of residual units. To maintain the output size the same as the size of input feature map, the same number of up-sampling and the max pooling blocks is applied. The attention mask in these attention modules can function as a feature selector during forward propagation as well as a gradient update filter during back propagation. The mask branches, in other words, can prevent incorrect gradients from

updating the trunk parameters. The RAN56 architecture is demonstrated in Figure S2. In this network, as going deeper through the layers, the attention-aware features from different attention modules change adaptively.

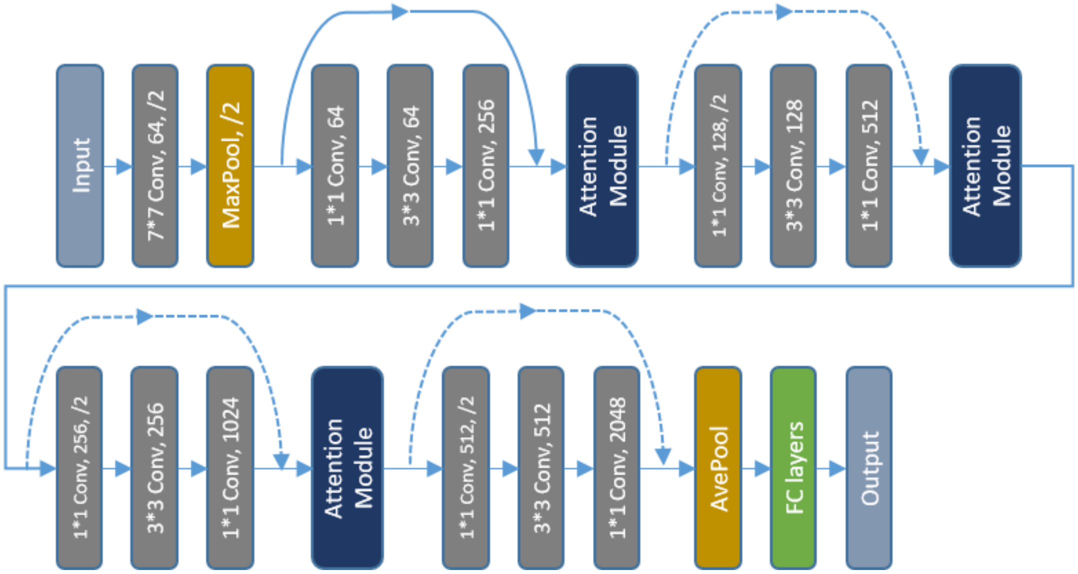

Figure S2. RAN56 architecture

References

[1] K. He, X. Zhang, S. Ren, and J. Sun, "Deep Residual Learning for Image Recognition," *Proc. IEEE Comput. Soc. Conf. Comput. Vis. Pattern Recognit.*, vol. 2016-December, pp. 770–778, Dec. 2015, doi: 10.1109/CVPR.2016.90.

[2] F. Wang *et al.*, "Residual Attention Network for Image Classification," *Proc. - 30th IEEE Conf. Comput. Vis. Pattern Recognition, CVPR 2017*, vol. 2017-January, pp. 6450–6458, Apr. 2017, doi: 10.1109/CVPR.2017.683.
